# Supplementary material for: Evidence for ion migration in hybrid perovskite solar cells with minimal hysteresis
Source: Nat Commun. 2016 Dec 22;7:13831. doi: 10.1038/ncomms13831 (PMC5192183; doi:10.1038/ncomms13831)
Supplement: Supplementary Information — Supplementary figures, supplementary tables, supplementary notes and supplementary references. [file ncomms13831-s1.pdf]

Supplementary Figures

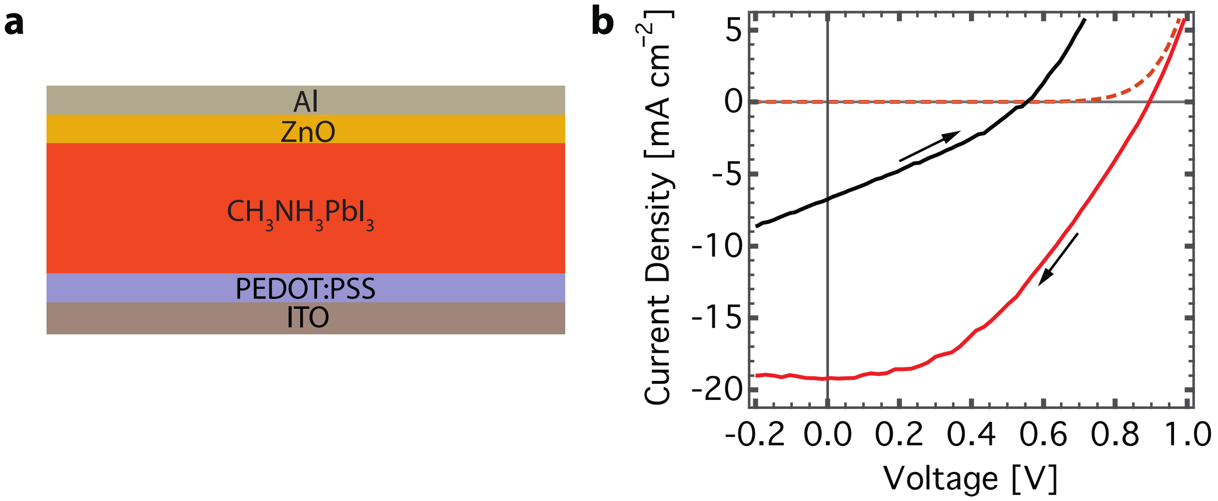

Supplementary Figure 1. Hysteresis in a top cathode cell using a ZnO electron collection layer instead of PCBM. (**a**) Device architecture for a ITO/PEDOT:PSS /$CH_3NH_3PbI_3$/ZnO/Al perovskite solar cell (**b**) Forward (black curve) and reverse (red curve) current-voltage scan (125 mV/s) exhibiting large *J-V* hysteresis (ITO/PEDOT:PSS /$CH_3NH_3PbI_3$/PCBM/Al devices showed minimal hysteresis *cf.* Fig. 1c). The ITO/PEDOT:PSS /$CH_3NH_3PbI_3$ stack of layers was fabricated as described in reference[1]. The $CH_3NH_3PbI_3$ layer was then contacted with a layer of ZnO nanoparticles. The 20 mg/ml solution of ZnO nanoparticles was prepared as described in reference [2], only in this instance the solvent was replaced completely with IPA. The nanoparticle solution was spin cast on top of the perovskite layer at 2000 rpm for 45 seconds followed by heating at 120 °C for 5 minutes. An evaporated Al layer was then deposited in the same way as for the PCBM containing device. A control ITO/PEDOT:PSS /$CH_3NH_3PbI_3$/PCBM/Al device was also prepared in which pure IPA was spun onto the PCBM layer and then heated prior to the evaporation of Al. The current-voltage sweeps for this control device do not exhibit hysteresis, similar to those shown in Fig.1c, and indicate that the IPA solvent and heating steps are not responsible for the presence of hysteresis in the device containing ZnO.

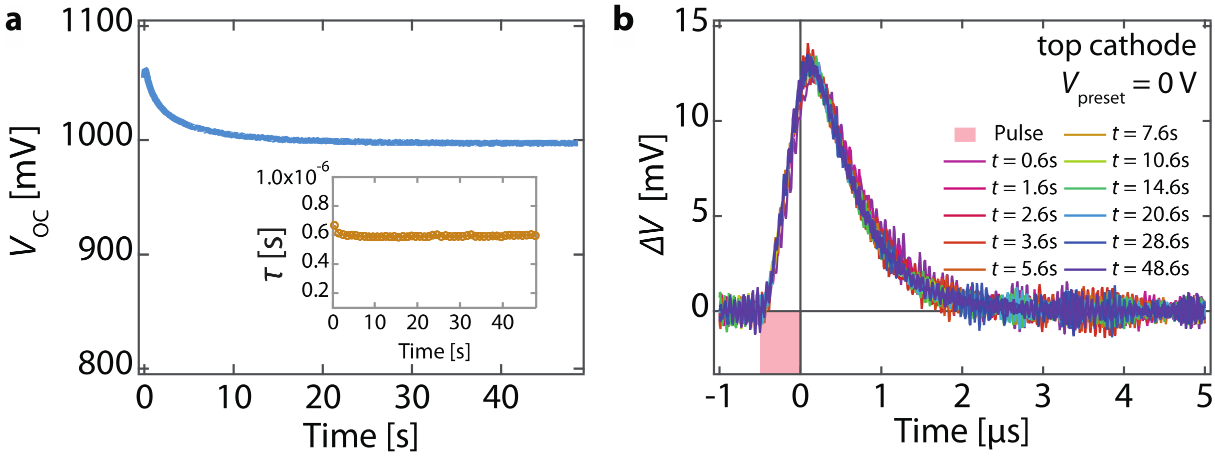

Supplementary Figure 2. Top cathode cell voltage transients of the transient measurements. (**a**) The evolution of the V_{oc} at one sun equivalent intensity following preconditioning with V_{preset} = 0 V in the dark. The inset shows the time constants, τ, for the single exponential fit to the photovoltage transient measurements made during the V_{oc} evolution. (**b**) The small perturbation transient measurements collected at different times during the V_{oc} evolution shown in (a). The shaded pink region shows indicates the time when the sample is illuminated by the laser excitation pulse in addition to the one sun equivalent bias light. Both the open circuit voltage and photovoltage transients remain relatively stable throughout the measurement, similar to when the cell is preconditioned at forward bias *cf*. Figs 3a and 3b.

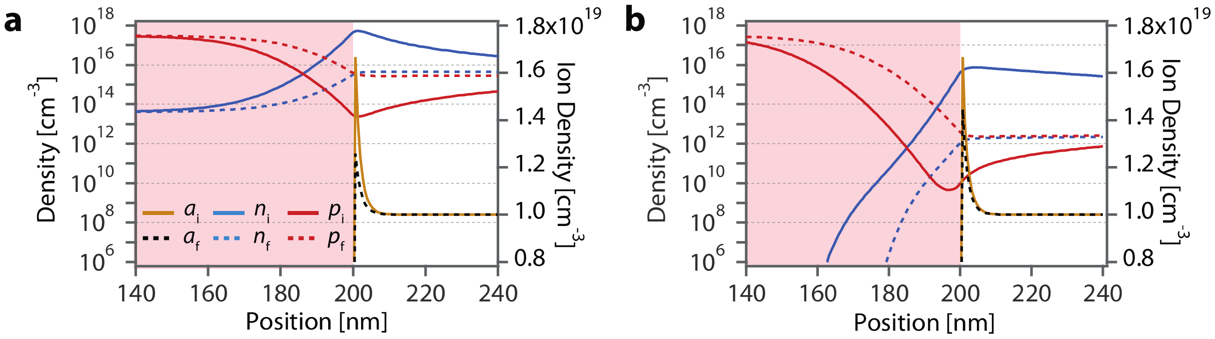

Supplementary Figure 3. Details of simulated charge density at interface. Details from the device simulation charge densities for (**a**) top cathode and (**b**) bottom cathode devices showing the interface between the p-type (shaded pink) and perovskite regions (at x = 200 nm), change in ionic charge profile and the depletion regions. The mobile ion, electron and hole densities are denoted by a, n and p respectively. Subscript i indicates the initial state of the cell following illumination and subscript f the final state after the V_{oc} has stabilised.

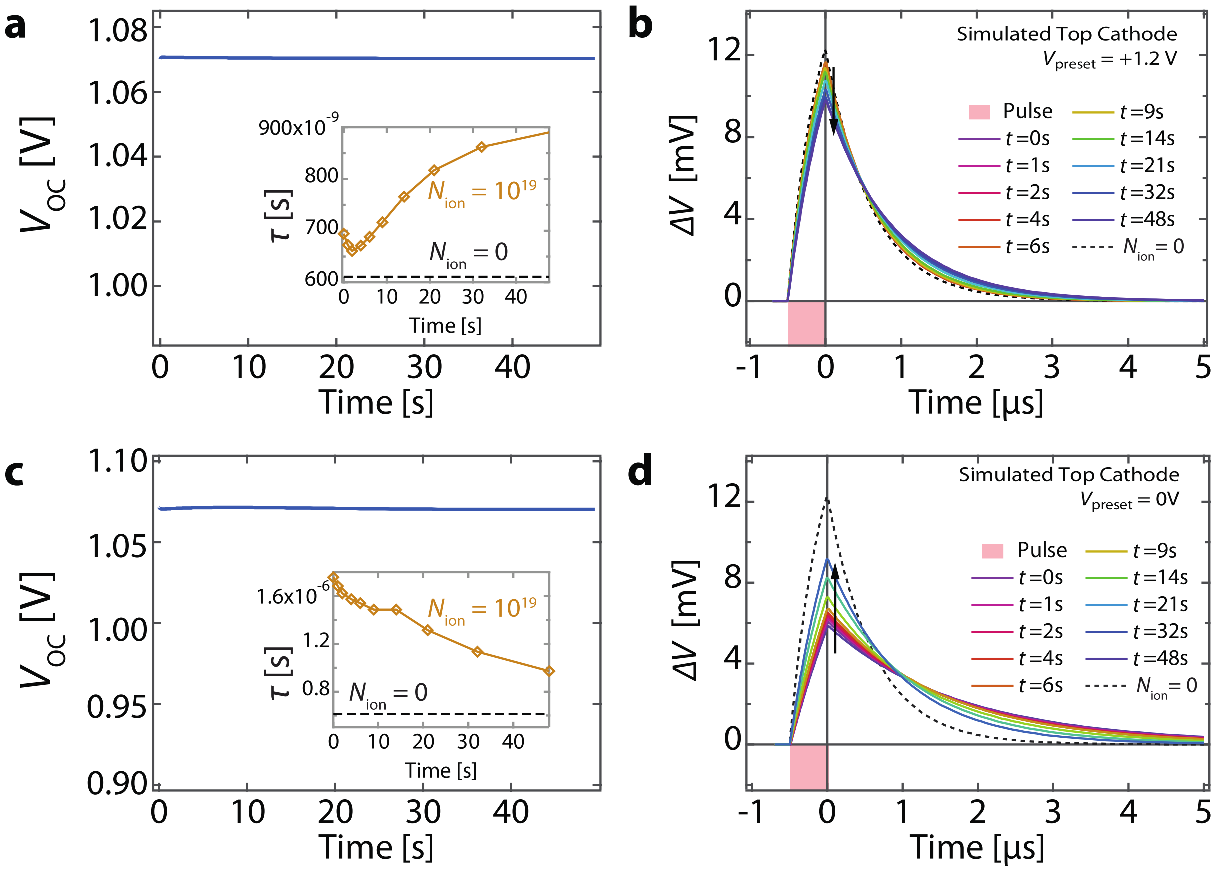

Supplementary Figure 4. Top cathode cell voltage transients of the transient simulations. Simulated transients of the transient photovoltage measurement for a device with the SRH recombination coefficient in the p- and n-type contact layers set to zero with (**a** and **b**) V_{preset} = +1.2 V, and (**c** and **d**) with V_{preset} = 0 V. The data is presented in the same way as that shown in Supplementary Fig. 2. A forward bias value of greater than the V_{oc} of the cell was used as in the experiment shown in Figs 1c and 3b. The small change in transient photovoltage magnitude observed in the simulated device could be attributed to the simplicity of using a p-i-n architecture and the absence of minority carrier accumulation at interfaces due to blocking contacts. An incorrect built-in voltage would also lead to the possibility of electronic charge not being able to fully screen the electric field created by ionic displacement after the cell has been switched to open circuit.

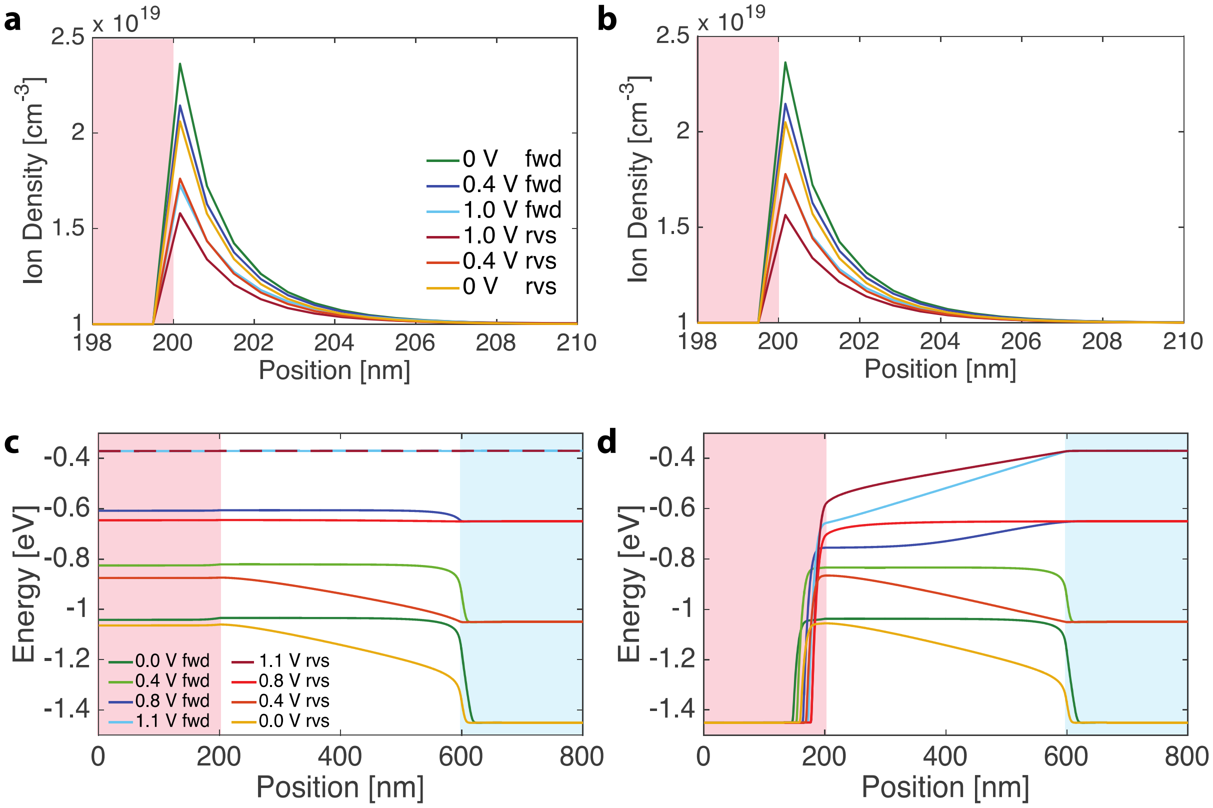

Supplementary Figure 5. Simulated ionic charge distributions and quasi-Fermi levels during *J*-*V* scans shown in Fig. 1. The distribution of mobile ionic charge is shown near the p-type/perovskite interface (at x = 200 nm) for the (**a**) top cathode device and (**b**) bottom cathode device. The p-type region is shaded pink. The corresponding electron quasi-Fermi levels are shown for the complete devices in (**c**) and (**d**) where the n-type region is shaded blue. The simulation outputs are shown for different applied voltage points (0, 0.4, 0.8 and 1.1 V) during the *J*-*V* scans at 40 mV s^{-1} from reverse to forward bias (fwd) and from forward to reverse bias (rvs).

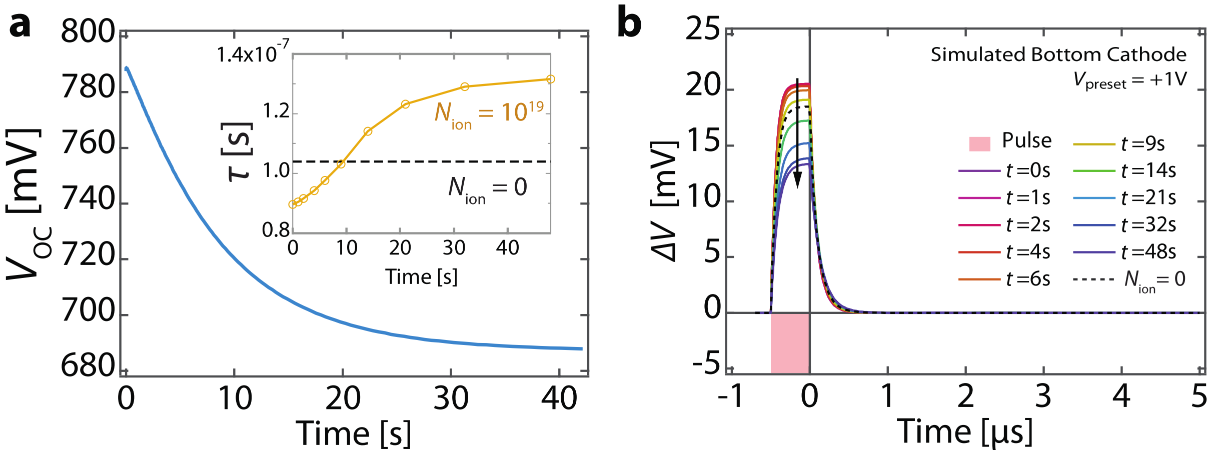

Supplementary Figure 6. **Bottom cathode cell voltage transients of the transient simulations with forward bias preconditioning.** Simulated transient of the transient photovoltage measurement for a bottom cathode device ($\tau_{n,SRH} = \tau_{p,SRH} = 2 \times 10^{-15}$ s, $N_{ion} = 10^{19}$ cm^{-3}) after preconditioning with V_{preset} = +1 V forward bias. See Figs 3c and 3d of the main text for experimental equivalent. (**a**) Shows the evolution of the background V_{oc} and the inset shows the evolution of the time constants (gold line, circles) of the small perturbation photovoltage transients shown in (**b**) where the pink shaded region represents the duration of the laser pulse. The dotted black line in the inset shows the time constants from an otherwise identical simulation but with no mobile ions (N_{ion} = 0 cm^{-3}). We note that the decay time constant after preconditioning with +1 V in the simulation is 1 to 2 orders of magnitude lower than observed experimentally. This could be attributed to an unrealistically high rate constant for band-to-band recombination or the absence of electron and hole blocking layers in the simulation, since no adjustment for possible variation in the built-in potential has been made.

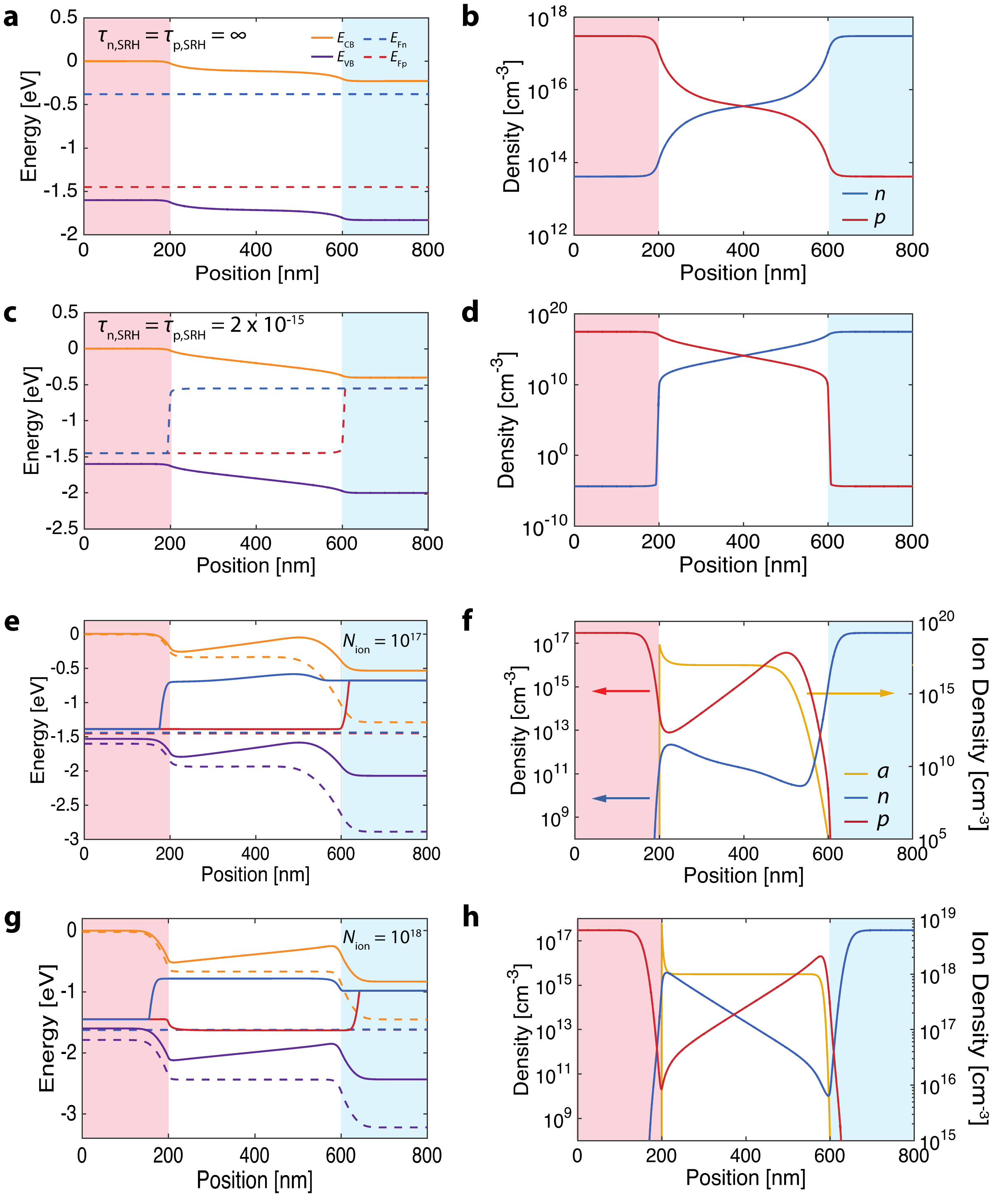

Supplementary Figure 7. Simulated effects of varying ion defect density and contact recombination. Band diagrams (left) and charge densities (right) for simulated p-i-n structure solar cells at open circuit with a uniform carrier generation profile of 2.5×10^{21} $cm^{-3}s^{-1}$. The p- and n-type regions are shaded pink and blue respectively. (**a**) and (**b**) Top cathode cell without mobile ion defects (N_{ion} = 0 cm^{-3}) or SRH recombination in the contacts ($\tau_{n,SRH} = \tau_{p,SRH} = \infty$ s). Standard quasi-Fermi level splitting is observed. (**c**) and (**d**) Bottom cathode cell without mobile ion defects (N_{ion} = 0 cm^{-3}) and with a high rate of SRH recombination ($\tau_{n,SRH} = \tau_{p,SRH}$ = 2 x10^{-15} s) in the contact regions. (**e**) and (**f**) Bottom cathode cell ($\tau_{n,SRH} = \tau_{p,SRH}$ = 2 x10^{-15} s) with 10^{17} cm^{-3} mobile ionic defects after the cell has equilibrated at short circuit (dashed

lines) and been switched to open circuit (solid lines) with generation on. While the ion density is large enough to screen out the field between approximately x = 210 nm and x = 440 nm, the asymmetry caused by a single ionic carrier type and associated giant Debye length at the perovskite/n-type interface results in the formation of a valley further from the contact. Although faster recombination is switched on in the contacts, a greater proportion of recombination takes place in the bulk compared to the cases in which N_{ion} = 10^{18} cm^{-3} and N_{ion} = 10^{19} cm^{-3}. This leads to a lower effective recombination rate constant, and resultant higher charge carrier densities and V_{oc}. We noted the V_{oc} was converging: Reducing the SRH recombination time constant by 2 orders of magnitude only reduced the initial V_{oc} (739 mV) by 16 mV suggesting that it would not be possible to reach the experimentally observed initial value (around 400 mV). Furthermore, a negative TPV could not be obtained using a simulated light pulse (generating an additional 2.5×10^{21} $cm^{-3}s^{-1}$ carriers). These results suggest that in the cells tested, > 10^{17} cm^{-3} ionic carriers are present. **(g)** and (**h**) Bottom cathode cell with 10^{18} cm^{-3} mobile ionic defects equilibrated at short circuit (dashed lines) and switched to open circuit (solid lines). In order to reach the experimentally observed V_{oc}, an increased SRH time constant of $\tau_{n,SRH} = \tau_{p,SRH} = 3\times10^{-16}$ s was required. However, all other results in this case remained similar to the case for N_{ion} = 10^{19} cm^{-3} due to shorter Debye lengths and effective screening of the built-in field.

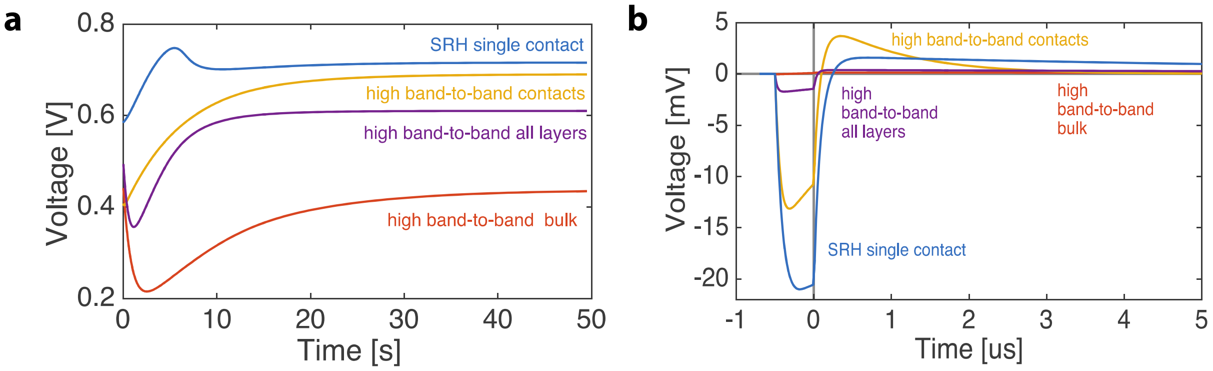

Supplementary Figure 8. Voltage transient simulations under different recombination schemes. (**a**) Slow open circuit voltage transients after the cell had reached equilibrium at short circuit (V_{preset} = 0 V) and **(b)** initial photovoltage transients (t = 0 s) for four different recombination schemes trialled in the study. See Supplementary Note 2 for details.

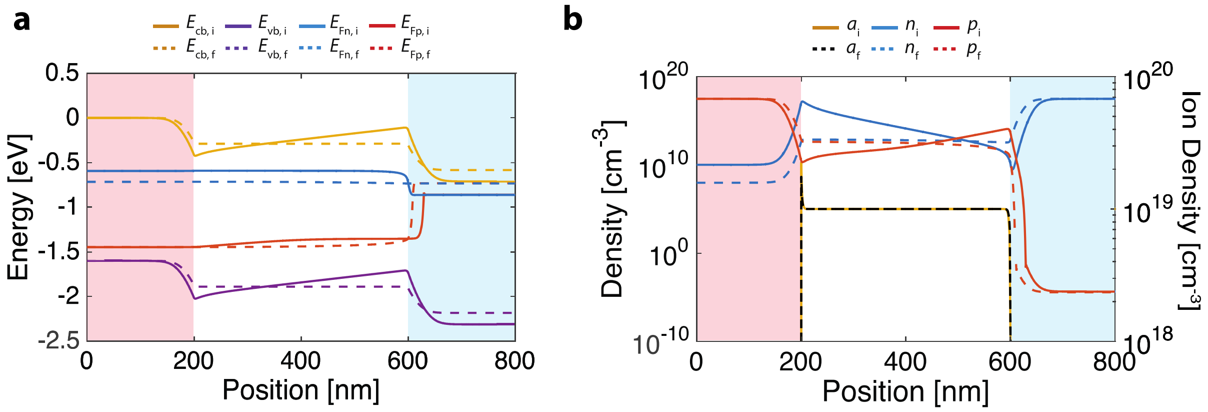

Supplementary Figure 9. Single contact recombination simulation data. (**a**) Band diagram and (**b**) charge densities for simulated p-i-n structure solar cells with a high SRH recombination rate coefficient ($\tau_{n,SRH} = \tau_{p,SRH} = 2 \times 10^{-15}$ s) in the n-type contact only. Solid and dashed lines indicate initial and final states respectively. The p- and n-type regions are shaded pink and blue respectively.

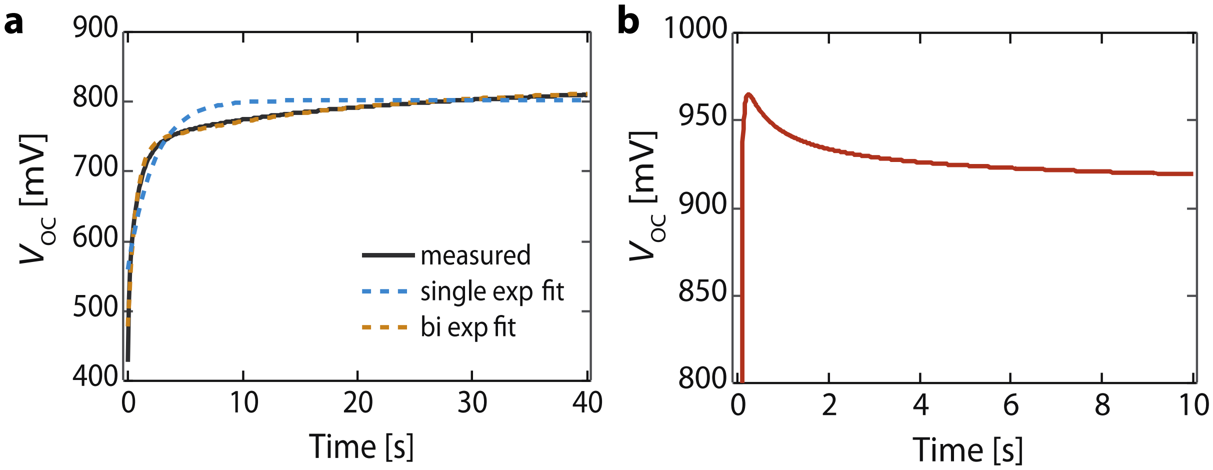

Supplementary Figure 10. Additional slow transient V_{oc} experimental measurements. Examples of biphasic increase in V_{oc} with time for (**a**) a bottom cathode device after preconditioning at short circuit for 60 seconds before switching to open circuit and (**b**) a top cathode device with the same measurement protocol. The characteristic hump in this case is suggestive of a higher rate of recombination in a single contact *cf*. Supplementary Fig. 8a.

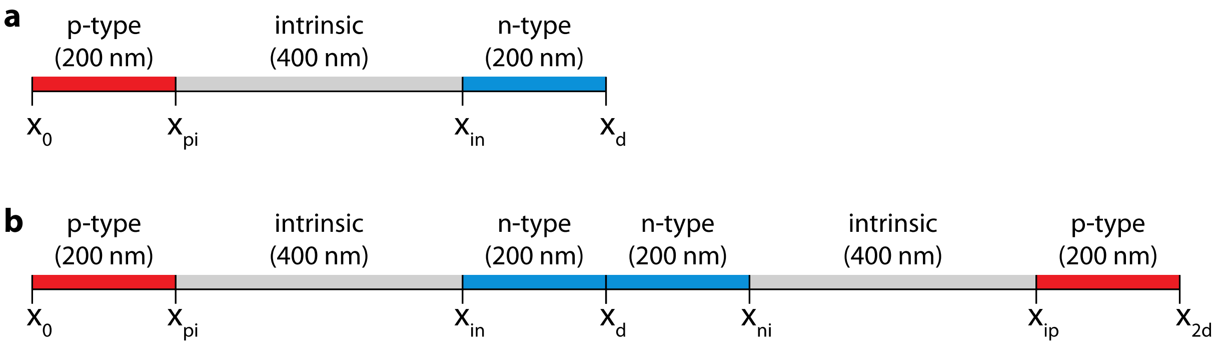

Supplementary Figure 11. Simulated device architectures. Device architectures with position labels and layer thicknesses for (**a**) the fixed potential boundary condition p-i-n (pin) simulation used for JV and current transient measurements, and (**b**) the symmetric p-i-n-n-i-p (spin) cell used for simulating open circuit voltage transients. The p-type, intrinsic and n-type regions are shaded red, grey and blue respsectively.

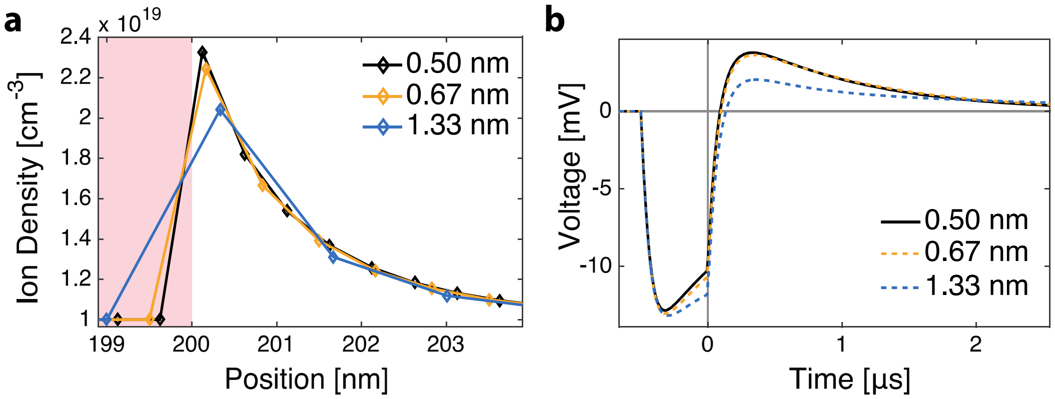

Supplementary Figure 12. Effect of mesh grid spacing on simulated solutions. Solutions for a bottom cathode device undergoing a transients of the transient measurement are shown for grid spacings of 0.5, 0.67 and 1.33 nm. (**a**) Ion accumulation region at the p-type/perovskite interface at equilibrium (where the p-type region is shaded pink) and (**b**) transient photovoltage simulations after the cell has been equilibrated at short circuit then switched to open circuit with different mesh spacings. A spacing of 1.33 nm per points leads to large errors in regions of high charge density gradients, for example at the p-type/perovskite interface (Supplementary Fig. 12a). The ion charge accumulation layer at this location has a characteristic length of a similar magnitude to the grid spacing (approximately 1.47 nm when the cell is at equilibrium) resulting in poor reproduction of the profile. These numerical inaccuracies are carried over to the transient photovoltage simulations (Supplementary Fig. 12b). A higher resolution grid spacing of 0.5 nm per point leads to marginally more accurate solutions compared with the 0.67 nm spacing used for the simulations in the main text. It is clear however that the solutions are convergent with respect to reduced grid spacing. A spacing of 0.67 nm was chosen for all other simulations presented in this study as a compromise between numerical accuracy and calculation time.

Supplementary Tables

Supplementary Table 1.   Key device simulation parameters.

Parameter name	Symbol	Value
Band gap[5]	E_g	1.6 eV
Built in voltage[6]	V_{bi}	1.3 V
Dielectric constant[7]	ε_s	20
Mobile ionic defect density[8]	N_{ion}	10^{19} cm^{-3}
Ion mobility	μ_a	10^{-12} cm^2 V^{-1} s^{-1}
Electron mobility[9]	μ_e	20 cm^2 V^{-1} s^{-1}
Hole mobility[9]	μ_h	20 cm^2 V^{-1} s^{-1}
Nominal band-to-band recombination coefficient	k_{btb}	10^{-10} cm^3 s^{-1}
p-type donor density	N_A	3.0 ×10^{17} cm^{-3}
n-type acceptor density	N_D	3.0 ×10^{17} cm^{-3}
p-type and n-type SRH time constants	$\tau_{n,SRH}$, $\tau_{p,SRH}$	2×10^{-15} s*
SRH trap energy, n-type	$E_{t,\ ntype}$	-0.2 eV (n-type)**
SRH trap energy, p-type	$E_{t,\ ptype}$	-1.4 eV (p-type) **
Effective density of states	N_0	10^{20} cm^{-3}
Generation rate	G	2.5×10^{21} cm^3 s^{-1}

All values for the perovskite phase are roughly based on literature values, except the band-to-band recombination rate, which was calculated based on a homogeneous charge carrier density of 10^{16} cm^{-3} and a nominal steady-state transient photovoltage decay time constant of 0.5 μs. The Shockley-Read-Hall (SRH) recombination rate coefficient and trap energies were chosen to reproduce the approximate open circuit voltage for the bottom cathode device. The generation rate was chosen to yield an absorbed photocurrent of ~ 16 mA cm^{-2}. The ionic mobility was adjusted from our previous literature estimate of about 4×10^{-11} cm^2 V^{-1} s^{-1} (ref. [8]) to a lower value of 10^{-12} cm^2 V^{-1} s^{-1} to correctly approximate the evolution time of the transient.

* SRH coefficient used in bottom cathode device only.

** In the band gap, below conduction band (0 V)

Supplementary Table 2. Table of Variables and symbols used for device simulation.

Variable name	Symbol	Units
Electron charge carrier density	n	cm^{-3}
Hole charge carrier density	p	cm^{-3}
Mobile ionic defect charge carrier density	a	cm^{-3}
Intrinsic carrier density	n_i	cm^{-3}
Static ionic defect density	N_{ion}	cm^{-3}
n-type electron equilibrium density	n_0	cm^{-3}
p-type electron equilibrium density	p_0	cm^{-3}
Electrostatic potential	V	V
Built in voltage	V_{bi}	V
Applied voltage	V_{app}	V
Current densities (electron, hole, ion defect)	$\mathbf{J}_n$, $\mathbf{J}_p$, $\mathbf{J}_a$	A cm^{-2}
Generation rate	G	cm^3 s^{-1}
Recombination rate	U	cm^3 s^{-1}
Net free charge density	ρ_f	cm^{-3}
Position	x	cm
Time	t	s

Supplementary Table 3. Constants and values used for device simulation.

Constant name	Symbol	Value (4 s.f.)*	Units
Boltzmann's constant	k_B	8.617×10^{-5}	eV K^{-1}
Temperature	T	300	K
Vacuum permittivity	ε_0	5.524×10^{5}	q^2 eV^{-1} cm^{-1}
Electron charge	q	1.619×10^{-19}	C

*Values in the simulation generally to higher accuracy.

Supplementary Notes

Supplementary Note 1. Derivation of zero dimensional kinetic model of recombination

$CH_3NH_3PbI_3$ is assumed to be an intrinsic semiconductor, the electron and hole concentrations [cm^{-3}] are approximated by Boltzmann statistics and given by:

$$n = N_{\mathrm{cb}} \exp \frac{E_{\mathrm{Fn}} - E_{\mathrm{cb}}}{k_{\mathrm{B}} T} \quad (1)$$

and

$$p = N_{\mathrm{vb}} \exp \frac{E_{\mathrm{vb}} - E_{\mathrm{Fp}}}{k_{\mathrm{B}} T} \quad (2)$$

where N_{cb} and N_{vb} are the effective density of states at the conduction band and valence edges which have energies E_{cb} and E_{vb} respectively. E_{Fn} and E_{Fp} are the electron and hole quasi-Fermi energies, k_{B} is Boltzmann's constant and T is the temperature.

The mean effective recombination rate, R, in the device is approximated as a power law

$$R = k(np)^{\gamma/2} \quad (3)$$

where k is the effective recombination rate constant [$cm^{3(\gamma-1)}$ s^{-1}]. If the concentration of electrons is equal to the concentration of holes ($n = p$) then this expression becomes:

$$R = kn^{\gamma} \quad (4)$$

where γ is the recombination reaction order. For example, if simple band-to-band recombination (bimolecular) is dominant then $\gamma = 2$, if Shockley-Read-Hall recombination via mid-gap states dominates then $\gamma = 1$.

In this study we measured the time constant, τ, of small perturbation transient decays. We wish to find the relationship between this measured values and the recombination rate constant, k. We follow the analysis in O'Regan et al.[3]: For a sufficiently small perturbation, the change in photovoltage is proportional to the change in carrier concentration in the device ($\Delta V_{\mathrm{oc}} \propto \Delta n$). Consequently, the small perturbation photovoltage decay time constant, τ, describes the rate at which the excess charge carriers decay:

$$\frac{\mathrm{d}\Delta n}{\mathrm{d}t} = -\frac{\Delta n}{\tau} \quad (5)$$

This can be described in terms of the total recombination rate using Supplementary Equation 4:

$$-\frac{\mathrm{d}\Delta n}{\mathrm{d}t} = k(n + \Delta n)^{\gamma} - kn^{\gamma} \approx kn^{\gamma}\left(1 + \gamma\frac{\Delta n}{n}\right) - kn^{\gamma} = k\gamma n^{\gamma-1}\Delta n \qquad (6)$$

Using Supplementary Equation 5 we can then write Supplementary Equation 6 as:

$$k \approx \frac{n^{1-\gamma}}{\gamma\tau} \qquad (7)$$

Given that the cell runs at quasi-steady state conditions we may state that the recombination rate is equal to the generation rate, $G = R$, then substituting Supplementary Equation 7 into Supplementary Equation 3 we can write:

$$G \approx \frac{(np)^{(1-\gamma)/2}}{\gamma\tau}(np)^{\gamma/2} = \frac{(np)^{1/2}}{\gamma\tau} \qquad (8)$$

Substituting the Supplementary Equations 1 and 2 into Supplementary Equation 8 gives:

$$G \approx \frac{(N_{\mathrm{cb}}N_{\mathrm{vb}})^{1/2}\exp\left[\frac{E_{\mathrm{Fn}} - E_{\mathrm{Fp}} + E_{\mathrm{vb}} - E_{\mathrm{cb}}}{2k_{\mathrm{B}}T}\right]}{\gamma\tau}$$

This can be rearranged to give an expression for the V_{oc} by finding the difference between E_{Fn} and E_{Fp}:

$$qV_{\mathrm{oc}} \approx E_{\mathrm{cb}} - E_{\mathrm{vb}} + 2k_{\mathrm{B}}T\log(\gamma\tau G) - k_{\mathrm{B}}T\log(N_{\mathrm{cb}}N_{\mathrm{vb}}), \qquad (9)$$

where in principle the only variables are γ, τ and G. During the transients of the transient experiment the bias light, and thus G is constant. If γ is also constant and the model were valid then the change in V_{oc} during the experiment would be given $2k_{\mathrm{B}}T\log(\tau)$.

Supplementary Note 2. Simulation details

Physical model

The device physics of the simulation is based on established semiconductor equations, detailed in Nelson[4] as described below using parameters and variables defined in Supplementary Tables 1–3.

Simulation coupled equations

The MATLAB pdepe 1-dimensional solver was used to solve the continuity equations and Poisson's equation for n, p, a and V as a function of time. The following relationships were used in the model.

Total current density

$$\mathbf{J} = \mathbf{J}_n + \mathbf{J}_p + \mathbf{J}_a + \mathbf{J}_{\text{disp}} \qquad (10)$$

Drift and diffusion current densities

$$\mathbf{J}_n = -q\mu_{\text{e}}(n\frac{dV}{dx} - k_{\text{B}}T\frac{dn}{dx}) \qquad (11)$$

$$\mathbf{J}_p = q\mu_{\text{h}}\left(p\frac{dV}{dx} + k_{\text{B}}T\frac{dp}{dx}\right) \qquad (12)$$

$$\mathbf{J}_a = q\mu_{\text{a}}\left(a\frac{dV}{dx} + k_{\text{B}}T\frac{da}{dx}\right) \qquad x_{\text{pi}} < x < x_{\text{in}} \qquad (13)$$

$$\mathbf{J}_a = 0 \qquad 0 < x < x_{\text{pi}} \ \& \ x_{\text{in}} < x < x_{\text{d}} \qquad (14)$$

Displacement current density

$$\mathbf{J}_{\text{disp}} = \varepsilon_0\varepsilon_{\text{s}}\frac{d}{dt}\left(\frac{dV}{dx}\right) \qquad (15)$$

Continuity equations

$$\frac{\partial n}{\partial t} = \frac{1}{q}\frac{\partial \mathbf{J}_n}{\partial x} + G_n - U_n \qquad 0 < x < x_{\text{d}} \qquad (16)$$

$$\frac{\partial p}{\partial t} = -\frac{1}{q}\frac{\partial \mathbf{J}_p}{\partial x} + G_p - U_p \qquad 0 < x < x_{\text{d}} \qquad (17)$$

$$\frac{\partial a}{\partial t} = -\frac{1}{q}\frac{\partial \mathbf{J}_a}{\partial x} \qquad x_{\text{pi}} < x < x_{\text{in}} \qquad (18)$$

$$\frac{\partial a}{\partial t} = 0 \qquad 0 < x < x_{\text{pi}} \ \& \ x_{\text{in}} < x < x_{\text{d}} \qquad (19)$$

Poisson's equation

$$\frac{\partial^2 V}{\partial x^2} = \frac{-q}{\varepsilon_0 \varepsilon_\mathrm{r}}(n - p + N_\mathrm{A}) \qquad 0 < x < x_\mathrm{pi} \qquad (20)$$

$$\frac{\partial^2 V}{\partial x^2} = \frac{-q}{\varepsilon_0 \varepsilon_\mathrm{r}}(n - p - a + N_\mathrm{ion}) \qquad x_\mathrm{pi} < x < x_\mathrm{in} \qquad (21)$$

$$\frac{\partial^2 V}{\partial x^2} = \frac{-q}{\varepsilon_0 \varepsilon_\mathrm{r}}(n - p - N_\mathrm{D}) \qquad x_\mathrm{in} < x < x_\mathrm{d} \qquad (22)$$

Intrinsic and Doped Carrier Densities

Intrinsic and doped equilibrium carrier densities were calculated using Boltzmann statistics. In order to generate the correct built-in potential at open circuit, doping densities in the *n* and *p*-type regions were calculated using the offset of the anode workfunction, ϕ_ano with the ionisation potential, ϕ_IP, and the cathode workfunction, ϕ_cat, with the electron affinity, ϕ_EA. For simplicity, the contacts are assumed to be Ohmic.

$$n_\mathrm{i} = N_0 \exp\left(\frac{-E_\mathrm{g}}{2k_\mathrm{B}T}\right) \qquad (23)$$

$$N_\mathrm{D} \approx n_0 = N_0 \exp\left(\frac{\phi_\mathrm{cat} - \phi_\mathrm{EA}}{k_B T}\right) \qquad (24)$$

$$N_\mathrm{A} \approx p_0 = N_0 \exp\left(\frac{\phi_\mathrm{IP} - \phi_\mathrm{ano}}{k_\mathrm{B} T}\right) \qquad (25)$$

Open circuit voltage

The open circuit voltage, V_oc is given by the difference in electron and hole quasi Fermi levels at the n-type and p-type boundaries of the device respectively:

$$V_\mathrm{oc} = V(x_\mathrm{d}) - V(x_0) + \frac{k_\mathrm{B}T}{q} \ln\left(\frac{n(x_\mathrm{d})\, p(x_0)}{n_\mathrm{i}^2}\right) \qquad (26)$$

Recombination

Band-to-band recombination, U_btb in our model uses a simplified for of bimolecular recombination:

$$\mathrm{U_{btb}} = k_\mathrm{btb}(np - n_\mathrm{i}^2) \qquad (27)$$

We used a reduced form of the Shockley-Read-Hall equation, where the capture cross section, mean thermal velocity, SRH coefficient and trap density are compressed into time constants, $\tau_{n,SRH}$ and $\tau_{p,SRH}$ for electrons and holes respectively.

$$U_{\mathrm{SRH}} = \frac{np - n_{\mathrm{i}}^2}{\tau_{\mathrm{n,SRH}}(p + p_{\mathrm{t}}) + \tau_{\mathrm{p,SRH}}(n + n_{\mathrm{t}})}, \quad (28)$$

where n_t and p_t are the electron and hole densities when the Fermi level is at the trap level.

p-i-n Structure Boundary Conditions

$$\mathbf{J}_n(0) = 0 \quad (29)$$

$$n(x_{\mathrm{d}}) = n_0 \quad (30)$$

$$p(0) = p_0 \quad (31)$$

$$\mathbf{J}_p(x_{\mathrm{d}}) = 0 \quad (32)$$

$$V(0) = 0 \quad (33)$$

$$V(x_{\mathrm{d}}) = V_{\mathrm{bi}} - V_{\mathrm{app}} \quad (34)$$

p-i-n-n-i-p Structure Boundary Conditions

$$\mathbf{J}_n(0) = 0 \quad (35)$$

$$\mathbf{J}_n(x_{2\mathrm{d}}) = 0 \quad (36)$$

$$\mathbf{J}_p(0) = 0 \quad (37)$$

$$\mathbf{J}_p(x_{2\mathrm{d}}) = 0 \quad (38)$$

$$V(0) = 0 \quad (39)$$

$$V(x_{2\mathrm{d}}) = 0 \quad (40)$$

Supplementary Note 3. Investigation of recombination schemes.

Implementing SRH recombination ($\tau_{n,SRH} = 10^{-17}$ s) in a single contact (n-type) allowed the negative TPV transient to be reproduced (Supplementary Fig. 8b blue trace) simulated using the transient of the transient (TROTTR) protocol (Fig. 2a). The simulated V_{oc} at time $t \approx 0$ was 200 mV higher than observed experimentally (Supplementary Fig. 8a blue trace) and converging with respect to decreasing recombination time constant. This was due to the charge carrier concentrations in the contacts reaching their doping density equilibrium values. A number of factors could lead to this discrepancy in V_{oc} such as lower-than-real rates of band-to-band recombination, a higher-than-real built-in voltage (which would alter the doping density in the contacts), and the uniform generation profile implemented in the simulation. The slow V_{oc} transient also exhibited a characteristic hump (Supplementary Fig. 8a blue trace) occasionally observed in top cathode V_{oc} transients (Supplementary Figure 10b). Implementing high rates of band-to-band recombination ($k_{btb} = 10^{-2}$ cm^{3}s^{-1}) in all layers of the cell also enabled reproduction of the negative TPV at early times in the TROTTR (Supplementary Fig. 8b purple trace). In this instance however, the slow V_{oc} transient exhibited an initial negative deflection of around 120 mV, after which it plateaued approximately 100 mV above its initial value (Supplementary Fig. 8a purple trace). Setting the bulk pervoskite band-to-band recombination rate to 10^{-1} cm^{3}s^{-1} allowed the experimental V_{oc} for the TROTTR experimental data to be obtained but neither the negative TPV nor slow V_{oc} transient were reproduced (Supplementary Figs 8a and 8b red traces). Finally, switching from high rates of SRH to band-to-band recombination in the contacts produced very similar results to when SRH was implemented in the main findings (Supplementary Figs 8a and 8b yellow traces).

Supplementary References

1 Jeon, N. J. *et al.* Solvent engineering for high-performance inorganic–organic hybrid perovskite solar cells. *Nat Mater* **13**, 897-903 (2014).

2 You, J. *et al.* Metal Oxide Nanoparticles as an Electron-Transport Layer in High-Performance and Stable Inverted Polymer Solar Cells. *Advanced Materials* **24**, 5267-5272 (2012).

3 O'Regan, B. C., Durrant, J. R., Sommeling, P. M. & Bakker, N. J. Influence of the TiCl4 treatment on nanocrystalline TiO2 films in dye-sensitized solar cells. 2. Charge density, band edge shifts, and quantification of recombination losses at short circuit. *Journal of Physical Chemistry C* **111**, 14001-14010 (2007).

4 Nelson, J. *The Physics of Solar Cells*. (Imperial College Press, 2003).

5 Leguy, A. M. A. *et al.* Experimental and theoretical optical properties of methylammonium lead halide perovskites. *Nanoscale* **8**, 6317-6327 (2016).

6 Schulz, P. *et al.* Electronic Level Alignment in Inverted Organometal Perovskite Solar Cells. *Advanced Materials Interfaces* **2**, 1400532 (2015).

7 Onoda-Yamamuro, N., Matsuo, T. & Suga, H. Dielectric study of $CH_3NH_3PbX_3$ (X = Cl, Br, I). *Journal of Physics and Chemistry of Solids* **53**, 935 - 939 (1992).

8 Eames, C. *et al.* Ionic transport in hybrid lead iodide perovskite solar cells. *Nat Commun* **6** (2015).

9 Leijtens, T. *et al.* Electronic Properties of Meso-Superstructured and Planar Organometal Halide Perovskite Films: Charge Trapping, Photodoping, and Carrier Mobility. *ACS Nano* **8**, 7147-7155 (2014).
